# Voluntary Exercise-Mediated Protection in TNBS-Induced Rat Colitis: The Involvement of NETosis and Prdx Antioxidants

**DOI:** 10.3390/antiox12081531

**Published:** 2023-07-30

**Authors:** Nikoletta Almási, Szilvia Török, Amin Al-awar, Médea Veszelka, László Király, Denise Börzsei, Renáta Szabó, Csaba Varga

**Affiliations:** 1 Department of Physiology, Anatomy and Neuroscience, University of Szeged, H-6726 Szeged, Hungary; tszilvia@bio.u-szeged.hu (S.T.); amin.al-awar@gu.se (A.A.-a.); veszmed@bio.u-szeged.hu (M.V.); denise@expbio.bio.u-szeged.hu (D.B.); szrenata@bio.u-szeged.hu (R.S.); vacs@bio.u-szeged.hu (C.V.); 2 Zala-Cereália Kft, H-8790 Zalaszentgrót-Tüskeszentpéter, Hungary; kiralyl@zala-cerealia.hu

**Keywords:** voluntary exercise, inflammation, peroxiredoxin, IBDS

## Abstract

Inflammatory bowel diseases (IBDs) are autoimmune disorders of the gut. It is increasingly clear that voluntary exercise (VE) may exert protection against IBDs, but the exact background mechanism needs to be elucidated. In the present study, we aimed to investigate the possible role of NETosis and the antioxidant peroxiredoxin (Prdx) enzyme family in VE-induced protection. Wistar Han rats were randomly divided into two groups: sedentary (SED) and VE. After the 6-week voluntary wheel running, animals were treated with 2,4,6-trinitrobenzene sulphonic acid (TNBS) as a model of colitis. Here, we found that VE significantly decreased inflammation and ulceration of the colon in the VE TNBS group compared with SED TNBS. We also found that VE significantly decreased the expression of protein arginine deiminase 4 (PAD4) and myeloperoxidase (MPO), and markedly reduced citrullinated histone H3 (citH3) compared with SED TNBS. Furthermore, VE caused a significant increase in the levels of Prdx6 in the control and TNBS groups. Taken together, we found that a prior 6-week VE effectively reduces inflammation in TNBS-induced colitis, and we suggest that the protective effect of VE may be mediated via the inhibition of NETosis and upregulation of Prdx6 antioxidant.

## 1. Introduction

Inflammatory bowel diseases (IBDs) are a group of gastrointestinal ailments characterized by inflammation and ulceration of the gut. The two main manifestations of IBD are Crohn’s Disease (CD) and ulcerative colitis (UC) [1]. The pathogenesis of IBD involves genetic predisposition, immunological characteristics, and environmental factors, such as the microbiome and diet [2]. Epidemiological studies revealed that genetic predisposition has a significant role in IBD. Several genes were found to be important in the context of IBD, such as the nucleotide-binding oligomerization domain containing 2 (NOD2) [3]. According to immunological characteristics, the pathogenesis of IBD comprises the dysregulation of innate and adaptive immune systems. Additionally, the importance of T cells, including T helper (Th)17 cells, has received particular attention recently [4]. Among immunological factors, several pro-inflammatory cytokines are important in the pathogenesis of IBD, such as tumor necrosis factor-α (TNF-α), interleukin-6 (IL-6), IL-17, and transforming growth factor-β (TGF-β) [5]. Furthermore, a Westernized diet, which comprises a low intake of fiber and high intake of fat and simple carbohydrates [6], and physical inactivity or sedentary lifestyle, also seem to contribute to the pathogenesis and exacerbation of IBD [7]. Several medical options are available to treat the symptoms of IBDs; however, alternative treatments, such as considering lifestyle changes and physical activity are also significant.

Sedentary lifestyle increases the risk of several diseases, such as cardiovascular diseases [8], type 2 diabetes mellitus [9], and cancer [10]. However, voluntary exercise (VE) seems to be an important tool against inflammatory conditions, since numerous beneficial features are described as a consequence of regular exercise [11]. Based on the myokine concept of exercise, its protective effect seems partly mediated through the increased myokine production of skeletal muscles during exercise [12]. Myokines are expressed and released into circulation through the contraction of skeletal muscles. Based on this recognition, it is increasingly clear that skeletal muscles can be considered as an endocrine organ [13]. Exercise induces the expression of numerous myokines, such as irisin, myostatin, IL-10, and IL-6 [14]. Interestingly, while acute physical activity induces an elevation in the pro-inflammatory IL-6 cytokine due to muscle functions, muscle-derived IL-6, as a myokine, has an anti-inflammatory effect modulated by circulating IL-6 receptors [15]. Regular exercise seems to exert protection against a vast number of diverse diseases, such as Alzheimer’s disease [16] and colorectal cancer [10], in addition to having anti-inflammatory effects [17]. Several options are available to model physical activity in rodents, such as swimming and running, with different methods (forced or voluntary). The benefits of forced activities are traceability and controllability; however, VE models are better for applying to human exercise [18]. It was found that 6-week forced exercise exacerbates, while 6-week VE attenuates, colonic inflammation in dextran sodium sulfate (DSS)-induced colitis in C57BL/6J mice [19]. A previous study of our laboratory also demonstrated that a 6-week VE significantly attenuated inflammation in another model of IBD [20]; however, the exact mechanism of this protection needs to be elucidated.

Neutrophils are the first defense against pathogens, and neutrophil accumulation can be seen locally at the site of the inflammatory damage [21]. Through their activation, neutrophils are the main cells responsible for neutrophil extracellular trap (NET) formation, also called NETosis, which leads to cell death during inflammation [22]. There are two main forms of NETosis, namely suicidal or classical and vital. Suicidal NETosis is nicotinamide adenine dinucleotide phosphate (NADPH) oxidase-dependent, lasts for a few hours, and causes the death of the neutrophils upon membrane rupture. On the contrary, vital NETosis is NADPH oxidase-independent, takes about 30 min, and neutrophils execute NET formation without membrane disintegration [23]. Both types of NETosis are crucial in host defense; however, excessive NET formation is considered a key factor in several autoimmune pathologies. Additionally, NETosis and its markers, such as myeloperoxidase (MPO), neutrophil elastase, protein arginine deiminase 4 (PAD4), and citrullinated histone H3 (citH3) are considered to be important in the pathogenesis of IBD [24]. PAD4 is a crucial constituent of this process, since it catalyzes chromatin decondensation in both types of NETosis [25]. Moreover, administration of Cl amide, a PAD4 inhibitor, effectively attenuated DSS-induced colitis and NET formation in mice [26].

Oxidative stress is also worth mentioning since there is an elevation in reactive oxygen species (ROS) production in IBDs, which leads to increased oxidative stress and inflammation [27]. Various mechanisms exist to counteract the detrimental effects of oxidative stress. The antioxidant system comprises mainly intracellular enzymatic antioxidants, intracellular non-enzymatic antioxidants, and extracellular antioxidants [28]. Peroxiredoxins (Prdx) are a group of intracellular antioxidant oxidoreductase enzymes with six members (Prdx1-6). Prdxs catalyze the elimination of hydrogen peroxide (H_2_O_2_). According to IBD research, four isoforms have emerged as being potentially involved in the pathogenesis of the disease, namely Prdx1 [29], Prdx2 [30], Prdx4 [31], and Prdx6 [32]. Furthermore, there is evidence for the importance of Prdxs in exercise-induced mechanisms [33], although the exact role of these enzymes is not fully known, and their functions are controversial.

Here, we aimed to investigate the protective effect of a prior 6-week VE in a TNBS-induced rat model of colitis. We hypothesized that the protective effect of VE may be mediated via the inhibition of NETosis and the elevation of the members of the Prdx antioxidant enzyme family.

## 2. Materials and Methods

### 2.1. Experimental Design and TNBS Instillation

All experiments were performed according to the standards of the European Community guidelines for the Care and Use of Laboratory Animals and were approved by the Institutional Ethics Committee (XX./4799/2015, 15 December 2015) at the University of Szeged.

**Figure 1:**
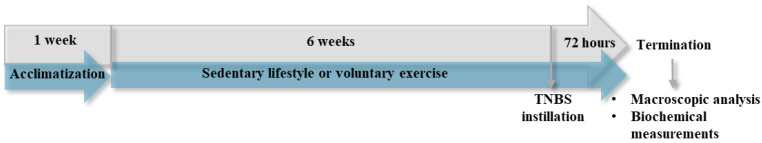
Experimental design.

Male Wistar Hannover rats (150–175 g, *n* = 55) were purchased from Toxicoop Ltd. (Dunakeszi, Hungary). Rats were housed in an animal facility with acclimatized temperature and 12 h day/night cycles. Food and water were given ad libitum throughout the experiment. After a week of acclimatization, animals were randomly divided into two groups: sedentary (SED) and voluntary exercise (VE). SED animals were housed in standard cages, while VE groups were held in a cage with free access to a running wheel for 6 weeks. After the 6 weeks, SED and VE animals were separated into three further groups: control (no treatment, n = 12), 50% EtOH (ethanol enema, n = 15), and TNBS (10 mg dissolved in 50% ethanol, n = 28). Colitis was induced following the method described by Morris et al. [34]. Briefly, animals fasted overnight, and an intracolonic (i.c.) TNBS enema was given through the anus with an 8 cm long polyethene tube under thiopental anesthesia (intraperitoneal (i.p.) 40 mg/kg). Rats had free access to the running wheel for the whole experiment. After 72 h of TNBS instillation, all animals were euthanized (thiopental, i.p. 100 mg/kg). The last 8 cm of the colon was removed from the rectum, gently opened, rinsed in physiological saline, and photographed with a compact camera. After the photographs were taken, the observed colonic regions were immediately frozen in liquid nitrogen. Before biochemical measurements, frozen tissues were powdered with a porcelain mortar and pestle. The samples were kept at −80 °C until measurements. The experimental design is illustrated in Figure 1.

### 2.2. Macroscopic Evaluation of the Lesions and Damage Score

The severity of macroscopically apparent inflammation and ulceration was analyzed by planimetry software which was developed in our laboratory (Stat_2_1_1, Szeged, Hungary). The pictures were evaluated in a randomized and blinded fashion. Macroscopically visible mucosal lesions were calculated and compared to the total studied 8 cm colonic segments. Results are expressed as a percentage.

The degree of colonic inflammation and ulceration was also evaluated in a randomized manner using a damage score [35] with slight modifications. This was scored as follows: 0, normal appearance; 1, focal hyperemia, no ulcers; 2, minor ulceration with inflammation at one site; 3, minor ulceration with inflammation at two sites; 4, major sites of damage approximately 1 cm along the length of the colon; 5–11, when major sites of damage ≥ 2 cm along the length of the colon, and the score was increased by 1 for each additional cm of involvement.

### 2.3. Western Blotting of TGF-β, citH3, PAD4, and MPO

Colonic tissues were measured and homogenized in RIPA buffer (Merck Millipore, Burlington, MA, USA) supplemented with phenylmethylsulfonyl fluoride (PMSF) (Sigma-Aldrich, Budapest, Hungary), 1/10 of the final volume. After homogenizing three times for 10 s each using an Ultrasonic Homogenizer UP-100H (Hielscher Ultrasonics, Teltow, Germany) on ice, homogenates were centrifuged at 15,000× *g* for 10 min at 4 °C. Supernatants were collected, and protein concentration was determined by using a Bradford assay. Based on the protein concentration, 50 µg from each sample was calculated and loaded onto 10% sodium dodecyl sulfate (SDS)-polyacrylamide gels (90 V, 2 h). Gels then were transferred to nitrocellulose membranes (2.5 h, 35 V). Membranes were dyed with Ponceau S and, after washing in TBS-T (pH 7.4), membranes were blocked in 5% milk or 5% BSA overnight. After blocking, blots were washed three times for 10 min in TBS-T and incubated with the first antibodies (room temperature, 2 h): anti-CitH3 (ab5103, Abcam, Cambridge, UK), anti-MPO (ab208670, Abcam, Cambridge, UK), anti-PAD4 (17373-1-AP, Proteintech, Manchester, UK), and anti-TGF-β (ab179695, Abcam, Cambridge, UK). Incubation with secondary anti-rabbit (DAKO Agilent, Santa Clara, CA, USA) or anti-mouse antibodies (DAKO Agilent, Santa Clara, CA, USA) conjugated with horseradish peroxidase was carried out for 1 h at room temperature. Bands were developed using an enhanced chemiluminescence system (ECL Plus, Amersham Pharmacia Biotech., Buckinghamshire, UK), and were analyzed using Quantity One Software version 4.5 (Bio-Rad Laboratories, Hercules, CA, USA). Each membrane was stripped and used for the detection of β-actin for normalization (ab20272, Abcam, Cambridge, UK; anti-mouse secondary antibody, DAKO Agilent, Santa Clara, CA, USA). Results were normalized to β-actin and presented as relative expressions.

### 2.4. Prdx ELISAs

For determination of Prdx1, -2, -4, and -6 in the colonic tissue, we used double-antibody sandwich ELISA kits. Prdx ELISAs (GA-E3965RT, GA-E3966RT, GA-E3968RT), GA-E3969RT) were purchased from GenAsia Biotech Co., Ltd. (Shanghai, China). Colonic tissues were homogenized two times for 10 **s** in phosphate buffer saline (PBS, pH 7.4), through the same homogenization procedure with Benchmark Scientific Handheld homogenizer D1000 (Benchmark Scientific, New Jersey, MA, USA). Homogenates were then centrifuged at 3000 rpm for 20 min at 4 ℃. Homogenization and sample preparation was carried out on ice. The levels of Prdxs were measured according to the manufacturer’s instructions, and optical densities (ODs) were measured at λ = 450 nm. Results are presented in ng/mg protein (Prdx1, Prdx2, Prdx4) or ng/µg protein (Prdx6).

### 2.5. Protein Concentration Determination

Protein concentration was determined via Bradford assay. Samples were diluted 25× with distilled water, and 20 µL of each sample was mixed with 980 µL distilled water for further dilution. Then, 200 µL of Bradford reagent was added to each sample. After gently mixing, samples were incubated for 10 min at room temperature protected from light. ODs were assayed at 595 nm with a spectrophotometer, and the results were compared to bovine serum albumin as a standard.

### 2.6. Statistical Analysis

All data are presented as mean ± SEM. Results of Western blotting were normalized to β-actin loading control. Statistical analysis was carried out with SigmaPlot 12 software, (Systat Software Inc., San Jose, CA, USA). Normality was checked in all datasets with the Shapiro–Wilk test. For parametric distribution, one-way ANOVA followed by the Holm–Sidak post hoc test was used, and the Kruskal–Wallis test followed by Dunn’s test was chosen in the case of nonparametric data. Differences were considered significant in all measurements when the *p* values were less than 0.05.

## 3. Results

### 3.1. Voluntary Exercise-Induced Protection on the Severity of Inflammation in TNBS-Induced Colitis

**Figure 2:**
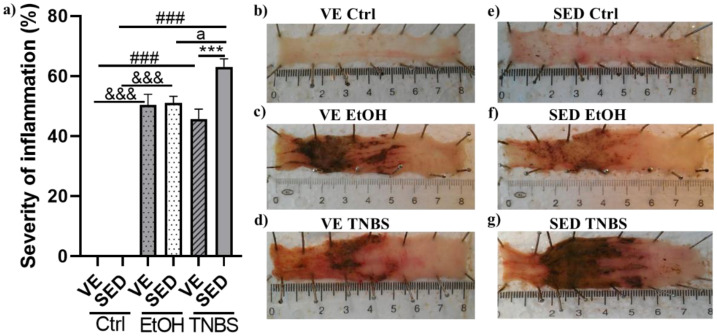
(**a**) Voluntary exercise (VE) significantly decreased inflammation of the colonic tissue in 2,4,6-trinitrobenzene sulphonic acid (TNBS)-induced rat colitis. SED: sedentary, Ctrl: control, EtOH: ethanol. Data are represented by mean ± SEM, n = 5–14/group. One-way ANOVA Holm–Sidak post hoc test. *** *p* < 0.001 SED vs. VE; ^###^ *p* < 0.001 ctrl vs. TNBS; ^&&&^ *p* < 0.001 ctrl vs. EtOH; ^a^ *p* < 0.05 EtOH vs. TNBS. Representative pictures of the colonic inflammation: (**b**) voluntary exercise control, (**c**) voluntary exercise EtOH, (**d**) voluntary exercise TNBS, (**e**) sedentary control, (**f**) sedentary EtOH, and (**g**) sedentary TNBS.

TNBS was used as a model of IBD, which represents the main symptoms of this ailment. In this study, the rats were placed in cages with free access to a running wheel for 6 weeks. Then rats received a single i.c. enema of TNBS to induce colitis. As shown in Figure 2a, in the SED groups, TNBS significantly increased colonic ulceration compared with the control group and with ethanol as such (63.072 ± 2.668 vs. 0 and 51.019 ± 0.296%). A similar tendency was observed in VE groups in the case of EtOH and TNBS compared with healthy running controls (50.393 ± 3.59 and 45.685 ± 3.357 vs. 0%). However, when comparing SED groups to VE, we found that VE significantly decreased the severity of inflammation in the VE TNBS group compared with SED TNBS (45.685 ± 3.357 vs. 63.072 ± 2.668%). Representative pictures of colonic tissues from the different groups are presented in Figure 2b–g.

### 3.2. Effects of Voluntary Exercise on the Damage Score of the Colonic Inflammation and Expression of TGF-β in TNBS Rat Colitis

**Figure 3:**
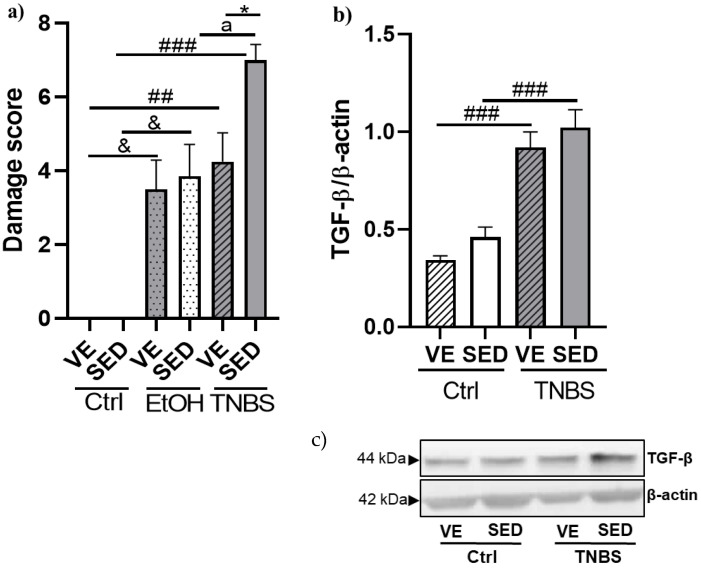
(**a**) Voluntary exercise (VE) significantly mitigated colonic score of inflammation and ulceration induced by 2,4,6-trinitrobenzene sulphonic acid (TNBS) in rats. (**b**) Effects of VE on the expression of transforming growth factor β (TGF-β) in TNBS-induced rat colitis. (**c**) Representative image of Western blot. SED: sedentary, Ctrl: control, EtOH: ethanol. Data are represented by mean ± SEM, n = 5–12/group. One-way ANOVA Holm–Sidak post hoc test. * *p* < 0.05 SED vs. VE; ^##^ *p* < 0.01 and ^###^ *p* < 0.001 ctrl vs. TNBS; ^&^ *p* < 0.05 ctrl vs. EtOH; ^a^ *p* < 0.05 EtOH vs. TNBS.

Colonic score damage was assessed in a randomized manner from the images, using a damage score with slight modifications. We found a significant elevation of ulceration in the colon after EtOH and TNBS administration. In the SED groups, there was a significant elevation in ulceration between EtOH and TNBS. Furthermore, we found a significant alleviation of colonic damage in the VE TNBS group compared with SED TNBS (Figure 3a).

A pro-inflammatory cytokine, TGF-β, was measured in our study by Western blotting. As shown in Figure 3b TNBS instillation significantly elevated the expression of the TGF-*β* cytokine in the SED and VE groups compared with each ctrl counterpart (1.022 ± 0.0915 vs. 0.461 ± 0.0503 relative expression; 0.920 ± 0.0794 vs. 0.343 ± 0.0205 relative expression). In the case of this cytokine, VE seemed to fail to decrease its elevated expression compared to the SED TNBS group.

### 3.3. The Effects of Voluntary Exercise on NETosis Marker Expressions in TNBS-Induced Colitis

**Figure 4:**
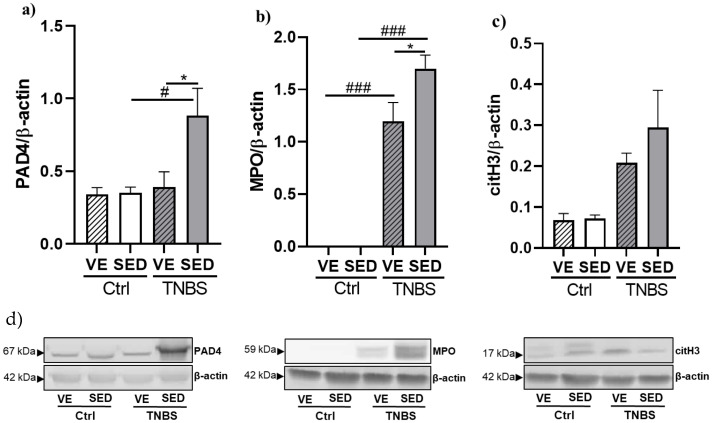
Voluntary exercise (VE)-induced attenuation of the expression of NETosis markers: (**a**) protein arginine deiminase 4 (PAD4), (**b**) myeloperoxidase (MPO), and (**c**) citrullinated histone H3 (citH3) in 2,4,6-trinitrobenzene sulphonic acid (TNBS) rat colitis. (**d**) Representative images of Western blot. SED: sedentary, Ctrl: control. Data are represented by mean ± SEM, n = 4–8/group. One-way ANOVA Holm–Sidak post hoc test. * *p* < 0.05 SED vs. VE; ^#^ *p* < 0.05 and ^###^ *p* < 0.001 ctrl vs. TNBS.

NETosis is a form of cell death which excessively occur in inflammatory conditions. Three markers of NETosis were measured by Western blotting in the current study. Figure 4a shows the elevated expression of PAD4 in the SED group after TNBS instillation compared with the control (0.883 ± 0.187 vs. 0.350 ± 0.0399 relative expression). VE, however, significantly decreased the expression of this protein compared with the SED counterpart (0.391 ± 0.105 vs. 0.883 ± 0.187 relative expression).

MPO expression was significantly increased after TNBS treatment compared with the control groups in the case of SED and VE as well (1.699 ± 0.129 vs. 0; 1.196 ± 0.180 vs. 0 relative expressions). Furthermore, a prior 6-week VE significantly diminished TNBS-induced inflammation and ulceration of the colon compared with the SED TNBS group (1.196 ± 0.180 vs. 1.699 ± 0.129 relative expression) (Figure 4b).

As shown in Figure 4c, the expression of the citH3 enzyme was markedly increased after TNBS treatment compared with SED or VE control groups. VE slightly moderated the elevated expression of citH3; however, the differences did not show statistical significance.

### 3.4. Effects of Voluntary Exercise on the Levels of Prdx Enzyme Family in TNBS Rat Colitis

**Figure 5:**
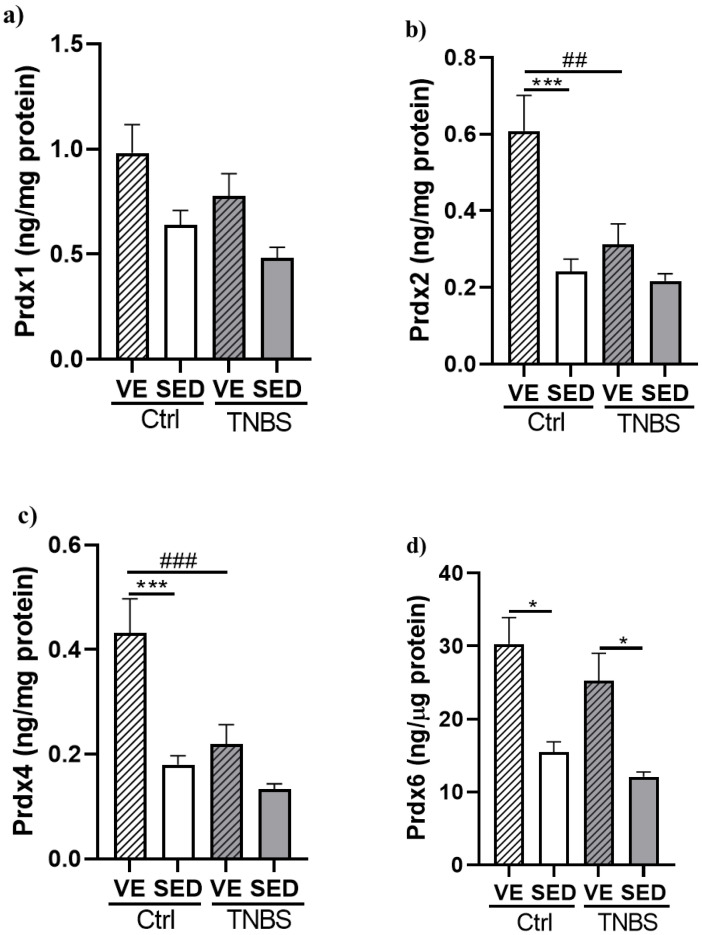
Effects of voluntary exercise (VE) on the levels of (**a**) peroxiredoxin-1 (Prdx1), (**b**) peroxiredoxin-2 (Prdx2), (**c**) peroxiredoxin-4 (Prdx4), and (**d**) peroxiredoxin-6 (Prdx6) in 2,4,6-trinitrobenzene sulphonic acid (TNBS)-induced rat colitis. SED: sedentary, Ctrl: control. Data are represented by mean ± SEM, n = 5–11/group. One-way ANOVA Holm–Sidak post hoc test or Kruskal–Wallis test followed by Dunn’s test. * *p* < 0.05 and *** *p* < 0.001 SED vs. VE; ^##^ *p* < 0.01 and ^###^ *p* < 0.001 ctrl vs. TNBS.

The levels of four isoforms of the Prdx enzyme family were measured in the current study with specific sandwich ELISAs. Prdx1 levels showed a slight decrease after TNBS treatment in the SED and VE groups compared to controls. Furthermore, VE slightly increased the levels of Prdx1 in both the control and TNBS groups, but the differences did not show statistical significance (Figure 5a).

Figure 5b shows that VE significantly increased the levels of Prdx2 isoform in the control animals compared with the SED control group (0.608 ± 0.093 vs. 0.241 ± 0.033 ng/mg protein). However, TNBS treatment significantly diminished the protective effect of VE compared with the VE control (0.311 ± 0.054 vs. 0.608 ± 0.093 ng/mg protein).

As shown in Figure 5c, the levels of Prdx4 significantly increased as a consequence of the 6-week VE compared with the SED control group (0.432 ± 0.065 vs. 0.180 ± 0.017 µg/mg protein). TNBS administration significantly reduced the levels of Prdx4 compared with VE control (0.219 ± 0.038 vs. 0.432 ± 0.065 µg/mg protein).

The levels of Prdx6 showed a significant elevation in the VE control group compared with the SED control group (30.291 ± 3.639 vs. 15.481 ± 1.402 ng/µg protein). Moreover, we found a significant increase in Prdx6 levels in the VE TNBS group compared with the SED TNBS (25.234 ± 3.795 vs. 12.071 ± 0.667 ng/µg protein) (Figure 5d).

## 4. Discussion

Here, we found that VE seems to have protective effects in TNBS-induced colitis in rats, since we showed that a prior 6-week VE effectively attenuated colonic inflammation and ulceration. We found that VE significantly diminished PAD4 and MPO NETosis markers, and markedly reduced citH3. Furthermore, we showed that VE significantly elevated Prdx2, -4 and -6 in the control rats compared to SED. Moreover, we showed a significant increase in the levels of the Prdx6 antioxidant due to a 6-week prior VE in the TNBS group compared to SED. Our results suggest that 6-week prior VE attenuates inflammation by inhibiting NETosis and enhancing the Prdx6 antioxidant.

IBD is considered a serious health issue, which has an increasing occurrence in developing countries. Several animal models are available to develop potential therapeutic options against IBDs. Of these, chemically-induced models are frequently applied based on their reproducibility and cost efficiency. DSS and TNBS are the most used agents to induce colitis in rodents. A single enema of TNBS causes serious inflammation and ulceration of the colonic tissue of rodents. TNBS efficiently reproduces the main symptoms occurring in CD patients, namely rectal bleeding, transmural inflammation, and a Th1/Th17 cytokine profile [36].

It is increasingly clear that the pathogenesis of IBD is associated with a Westernized lifestyle and physical inactivity [7]. Physical inactivity is a risk factor for various diseases; however, the effects of exercise are controversial, since numerous research showed that its effects depend on the type of exercise. As an example, it was found by Cook et al. [37] that forced exercise seems to promote pro-inflammatory pathways while VE is protective in DSS-induced colitis in C57Bl/6J mice. In accordance with them and with our previous work [20], our current results showed that prior 6-week VE significantly attenuated inflammation and ulceration in TNBS-induced rat colitis. Additionally, in a DSS-induced chronic colitis study, Qin et al. [38] found that swimming for 7 weeks (1 or 1.5 h for 5 days/week) significantly attenuated colonic injury and spleen enlargement in Sprague Dawley rats. Based on the measured parameters, they suggest that the anti-inflammatory effect of swimming is mediated via the attenuation of pro-inflammatory factors, oxidative stress, and apoptosis. These findings correlate with ours on the beneficial effects of regular exercise.

Furthermore, we found an elevated level of TGF-β cytokine in TNBS-treated groups compared to controls. In accordance with our results, Qui et al. [39] showed that TNBS treatment significantly elevated TGF-β expression in rats, and Del Zotto et al. [40] found the same alteration in the colonic tissue of IBD patients. Furthermore, Silva et al. [41] found that chronic exercise significantly attenuated TGF-β levels in middle-aged obese mice. However, in the current study we found no significant difference in the controls or TNBS groups between SED and VE.

NETosis is a type of cell death that occurs in inflammatory conditions. Through this process, neutrophils form a web-like structure of NET from DNA, histones, fibers, and neutrophil granular proteins [23]. NETosis is significantly elevated in IBD, thus, providing a potential therapeutic target [24]. In this study, we found a significant increase in PAD4 and MPO expression and a marked increase in the expression of citH3 after TNBS treatment compared with the SED control. Zhang et al. [42] and the previous work of our laboratory [43] showed the same alteration in NETosis markers after TNBS administration. Moreover, here we found that the prior 6-week VE significantly attenuated PAD4 and MPO expression and markedly reduced the expression of citH3 compared with the SED TNBS group. Thus, we suggest that VE seems to reduce NET formation partially via decreasing NETosis markers. Orysiak et al. [44] found in young male athletes that intensive physical training tends to decrease neutrophil counts in the blood while increasing NET formation. Based on their findings, NETosis seems to be important in muscle function and local inflammatory processes during intensive exercise. In contrast, in our study, the different type of activity, VE, caused a significant decrease in NETosis markers compared to the TNBS group. Our results correlate with the findings of Shi et al. [45], who showed that 5-week treadmill running significantly decreases lung inflammation and NET formation in LPS-induced acute lung injury in mice.

Oxidative stress is a major contributor to the pathogenesis of IBD, thus, strengthening the antioxidant mechanisms is crucial in IBD patients [46]. It is increasingly clear that the level of H_2_O_2_ is important to muscle adaptation to exercise. Prdx enzymes are a class of oxidoreductases that seems to have a role in adaptation to exercise via catalyzing and, thus, regulating H_2_O_2_ levels [33]. Here, we found that, in control animals, VE significantly elevated the levels of Prdx2, Prdx4, and Prdx6; however, we found a significant increase only in the levels of Prdx6 when comparing the SED and VE TNBS groups. Moghaddam et al. [47] found significantly up-regulated Prdx2 levels in erythrocytes of type 2 diabetes patients that regular physical exercise, while not affecting Prdx1 levels. Their findings correlate with ours considering the effect of VE-induced alterations in Prdx1 and Prdx2 in the case of the control animals; however, VE did not cause a significant change when comparing SED and VE TNBS groups. Richters et al. [48] found in cardiomyocytes that Prdx1 and Prdx2 did not change, while Prdx4 and Prdx6 decreased significantly due to regular physical exercise in wild-type mice. These findings partially correlate with ours, since we also found that VE did not alter Prdx1 significantly in the control animals; however, we found a significant increase in the levels of Prdx2, Prdx4, and Prdx6 between the control groups due to VE.

Our current results show a beneficial effect of VE against TNBS-induced colitis; however, further investigations are needed. Future studies should focus on the different modalities of exercise, such as timing, duration, and intensity. Additionally, exercise should also be validated in human clinical trials for its possible application as a therapeutic intervention or prevention against inflammatory diseases, including IBDs.

## 5. Conclusions

In conclusion, we found that a prior 6-week VE efficiently attenuates colonic inflammation and ulceration in TNBS-induced rat colitis. Furthermore, our results suggest that VE-induced protection is mediated by inhibiting NETosis and enhancing Prxd6 antioxidants. Therefore, our current results show further evidence for the beneficial effects of regular exercise against IBDs and contributes to a better understanding of the mechanisms behind VE-induced protection.

## Data Availability

The datasets generated and analyzed in this study are available upon reasonable request from the corresponding author.
